# Link prediction in complex network using information flow

**DOI:** 10.1038/s41598-023-41476-9

**Published:** 2023-09-05

**Authors:** Furqan Aziz, Karin Slater, Laura Bravo-Merodio, Animesh Acharjee, Georgios V. Gkoutos

**Affiliations:** 1 School of Computing and Mathematical Sciences, University of Leicester, University Rd, Leicester, LE1 7RH UK; 2 Institute of Cancer and Genomic Sciences, University of Birmingham, Birmingham, B15 2TT UK; 3 Institute of Translational Medicine, University of Birmingham, Birmingham, B15 2TT UK; 4 NIHR Surgical Reconstruction and Microbiology Research Centre, University Hospital Birmingham, Birmingham, B15 2WB UK; 5 MRC Health Data Research UK (HDR UK), London, UK; 6 NIHR Experimental Cancer Medicine Centre, Birmingham, B15 2TT UK; 7 Centre for Health Data Science, Birmingham, B15 2WB UK; 8 Centre for Environmental Research & Advocacy, University of Birmingham, Birmingham, B15 2TT UK

**Keywords:** Computational biology and bioinformatics, Mathematics and computing

## Abstract

Link prediction in complex networks has recently attracted a great deal of attraction in diverse scientific domains, including social and biological sciences. Given a snapshot of a network, the goal is to predict links that are missing in the network or that are likely to occur in the near future. This problem has both theoretical and practical significance; it not only helps us to identify missing links in a network more efficiently by avoiding the expensive and time consuming experimental processes, but also allows us to study the evolution of a network with time. To address the problem of link prediction, numerous attempts have been made over the recent years that exploit the local and the global topological properties of the network to predict missing links in the network. In this paper, we use parametrised matrix forest index (PMFI) to predict missing links in a network. We show that, for small parameter values, this index is linked to a heat diffusion process on a graph and therefore encodes geometric properties of the network. We then develop a framework that combines the PMFI with a local similarity index to predict missing links in the network. The framework is applied to numerous networks obtained from diverse domains such as social network, biological network, and transport network. The results show that the proposed method can predict missing links with higher accuracy when compared to other state-of-the-art link prediction methods.

## Introduction

The field of complex networks has gained popularity in recent years as a tool for analysing complex systems. Researchers from diverse scientific domains have used complex networks to model complex systems, helping them to analyse and address real world problems ranging from technological to biological networks^1,2^. The main advantage of using a complex network lies with its ability to allow us to understand and further analyse the behaviour and the characteristics of the whole system. Such characteristics are typically observed once the individual entities of the system are put together to form the system and cannot be inferred from the properties of individual entities. For example, once a complex system is modelled as a complex network, we can gain a better understanding of the evolution of the network as well as of how the information flows from one entity to the other entity within the complex system. Let us assume that individuals within a population are modelled as nodes and that two nodes are connected via a link if the two individuals have been in contact with each other. Such a modelling scenario could, for example, aid the forecast of the disease spread in a contact network using interaction information between the individuals in the network.

One of the most important problems in network science lies in the prediction of missing relationships within a network. This problem is challenging for two primary reasons. Firstly, the process of acquiring all existing relationships between all possible pairs of nodes is not easily achievable and usually results in many missing links in the network. Secondly, there are pairs of individuals in the network that are not connected when the data was acquired but are highly likely to connect in the near future. For example, in a social network, such as Facebook, there is always a possibility of individuals forming new friends in future. This process of identifying missing or yet unformed links is commonly referred to as link prediction^3^. Link prediction is important both from a theoretical, as well as a practical, perspective. In terms of the former, the problem of link prediction is important due to its ability to help us understand how the network grows and evolves with time. For example, in a contact network, link prediction can help us understand the spread of a disease in the network. From a practical perspective, link prediction can be directly applied to real-world networks to predict missing links. For example, in a protein-protein interaction network, link prediction can help us identify missing links between two proteins that will save resources and costs related to expensive in-vivo and in-vitro experiments.

The importance of link prediction has inspired many researchers from various scientific disciplines to develop novel link prediction algorithms that can not only be applied to a particular problem for which it is developed but could also be generalised to networks obtained from other domains. This follows because networks representing different complex systems usually share common topological features that are not present in regular or completely random networks^4^. For instance when a social network grows, two disconnected nodes that are close to each other in terms of shortest path distance are more likely to get connected by a link as compared to the nodes that are many hops away from each other. Therefore, many real-world networks tend to form clusters. This is generally not true for random networks where every pair of nodes is equally likely to be connected by a link. This allows us to use the properties of a given real-world complex network to predict which pair of disconnected nodes is more likely to get connected in the near future. These properties could either be the local properties that only consider the immediate neighbours of the two query nodes or the global properties that take into account the information about a larger subset of the nodes of the network to predict the missing link between the two nodes.

In this paper we develop a novel framework that is linked to a diffusion process on a network. Given two disconnected nodes, the proposed method considers both the neighbourhood structure of the nodes and their relative positions in the network to estimate the likelihood of a link between the two nodes. Our framework is based on the parametrised matrix forest index (PMFI)^5^ and the resource allocation (RA) index^6^. We demonstrate how the PMFI is linked to the flow of heat in the network. When combined with the RA index, we show the PMFI for small values of $\beta$ can predict links with higher accuracy and also respects the community structure of the input network. We apply the proposed framework to numerous datasets obtained from diverse domains and empirically show that it improves the performance of both the local and the global similarity indices.

## Related work

The problem of link prediction has been extensively studied over the last two decades and many efficient approaches have been proposed to predict the connection probability between two disconnected nodes in a given complex network. One of the most popular categories of link prediction algorithms is the structural-based similarity index which renders topological structural properties of the network along with the features of individual nodes to estimate the likelihood of existence of a link between two nodes that are not already connected in the network. One of the most widely used structural-based similarity index is the common neighbour (CN) index^7^. This index is based on the idea that two strangers, sharing a common friend, may be introduced by that friend. Although originally developed for social networks, CN has been proved successful in predicting links in other real-world networks, such as biological networks. One of the most powerful features of CN lies with its easy implementation, as well as its high computational efficiency, resulting in reasonably good performance over many real networks.

One of the limitations of the CN index is that it only considers the local topology of the underlying network and ignores the features of individual nodes and their neighbours. For instance, while computing the CN index, the degrees of individual nodes and their neighbours are not considered. This means that two different neighbouring nodes with different degrees will contribute equally to the CN score of the node. To overcome this problem a number of variations have been proposed to achieve higher performance on specific datasets. Adamic-Adar (AA) index^8^ is one of the earliest attempts to improve the performance of CN index. It is defined as the sum of the inverse logarithmic degrees of the common neighbours of the two nodes. This definition is based on the concept that common neighbours with large neighbourhoods are less significant when predicting a connection between two nodes compared with common neighbours with small neighbourhoods. A different, albeit relevant, index is the resource allocation (RA) index^6^ which is defined as the sum of inverse degrees of the common neighbours of the two nodes. The RA index performs better than the AA index on many real-world networks^9^. Another commonly used similarity measure that could be defined in terms of CN index is the Jaccard Coefficient^10^. It measures the proportion of common neighbours over the total number of neighbours achieving its maximum if all neighbours are common to both vertices. An alternative approach to CN-based measures is the popular Preferential attachment (PA) index^11^ that is motivated by the popular Preferential attachment principle underlying the evolution of scale-free networks. This index ignores the common neighbours of the two query nodes, depending only upon the degrees of the individual nodes. The more connected a node is, the more likely it is to receive new links.

The similarity indices discussed so far fall in the category of link prediction methods that are termed as local link prediction indices since these indices make use of the properties of the nodes or their immediate neighbours. From a practical point of view, local similarity indices are very effective as they are easy to implement and have the ability to predict most true links between nodes that are reasonably close to each other in the network in terms of shortest path distance. However, since these methods are based on a very small subset of the network, they may fail to predict important missing links in the network. Global link prediction algorithms form an alternative to predict missing and future links that aiming at improving the performance of local link prediction algorithms by considering network-level information, such as node centrality^12^, shortest path distance^13^, and random walks^14^ that are computed using a larger subset of nodes of the network. Global link prediction methods generally outperform local methods since they consider the whole topology of the network and can potentially predict links between any possible pairs of disconnected nodes in the network, even if these pairs are more than one hop away from each other. These methods are also consistent with the evolution of the network in the sense that they maintain the statistical properties of the network when new links are added^15^. However, global methods are generally more expensive to compute. Katz index^16^ forms an example of such methods considering the set of different length paths between two nodes whose connection probability has to be determined. It is based on the idea that two disconnected nodes are more likely to connect if there are more paths of length greater than one connecting the two nodes. The CN index could be considered a special case of Katz index that only takes into account paths of length 2. Another very popular global similarity index is the matrix forest index that estimates the likelihood of existence of a link between two nodes by finding the number of spanning rooted forests consisting of both the nodes where either the first or second node is considered a root. A similar group of global similarity indices is based on random walks on the network. These include indices such as average commute time^14^, random walk with restart (RWR) which is a direct application of the PageRank algorithm^17^, and superposed random walk (SRW)^18^. Finally, matrix completion methods are another common group of methods that convert the adjacency matrix of the network to a likelihood matrix. The link prediction problem is then transformed into an optimisation problem for the likelihood matrix^19^.

It is worth noting that, although the global similarity indices typically result in superior performance, when compared to local similarity indices, in some cases, a local similarity index may outperform a global similarity index. This is due to the fact that complex networks obtained from different domains evolve differently despite the fact that they share many common structural properties. Therefore there is not a single similarity index, or a category of similarity indices for that matter, that could be applied to all types of networks. In recent years, a number of attempts have been made to develop link prediction algorithms that combine the strengths of both the local link prediction methods and the global link prediction methods. One such category is the Quasi-local link prediction algorithm that provides a trade-off between accuracy and the computational time between the local and the global link prediction algorithms. For example, Lü et al. have defined the local path (LP) index^20^ that extends the CN index to paths of length 3 and can be considered as a special case of Katz index. The authors have demonstrated that the LP index is not only computationally efficient but also results in reasonably good performance when compared to Katz index. A more powerful approach is to incorporate the information of nodes that are one hop away from the query nodes^21^. Moreover, Kovács et al.^22^ have proposed an index that is based on paths of length three and have shown its applications in prediction of protein interactions. On the other hand, Cannistraci et al.^23^ have shown how the performance of local link prediction algorithms could be improved by incorporating the local community structure of the network. The resulting framework proved useful in predicting missing links in brain networks. Finally, in one of our recent works^9^, we have developed a suite of methods for extending local similarity indices to their global and quasi-local counterparts. We later extended this work to bipartite networks^24^ where we have explored its applications in predicting multimorbidity in elderly patients with multiple chronic conditions.

## Methods

We first examine some fundamental definitions and concepts related to network representations as well as to network heat diffusion processes that will aid our understanding of the theoretical underpinnings of our proposed framework.

### Preliminaries

A network consists of a finite non-empty set of nodes and a set of connections between nodes called links. A weighted network is a network whose links (or nodes) are assigned real values called the weight of the link (or node). In this paper we only consider link-weighted networks. A network is usually represented by a square matrix whose size is equal to the number of nodes in the network. The most commonly known representation is the adjacency matrix of the network that is used to represent both the weighted and the unweighted networks. For a weighted graph, the (*i*, *j*)-th entry of the matrix is equal to the weight of the (*i*, *j*)-th link $w_{ij}$, if node *i* is connected to node *j*:

1 $$\begin{aligned} A(i,j)={\left\{ \begin{array}{ll} w_{ij} &{} \quad \text{ if }\, (i,j)\in E,\\ 0 &{}\quad \text{ otherwise. } \end{array}\right. } \end{aligned}$$

For an unweighted graph the weights $w_{ij}$ are set to a constant value. Another very useful representation for a network is the Laplacian matrix which is defined as $L=D-A$. Here *D* is a diagonal matrix whose *i*-th diagonal entry represents the degrees of the *i*-th node of the network. For a weighted network, it is defined as follows:

2 $$\begin{aligned} L(i,j)={\left\{ \begin{array}{ll} -w_{ij} &{} \quad \text{ if }\, (i,j)\in E,\\ \sum _{(i,k)\in E}w_{ik} &{} \quad \text{ if }\, i=j,\\ 0 &{} \quad \text{ otherwise. } \end{array}\right. } \end{aligned}$$

The Laplacian matrix is sometimes also referred to as discrete Laplacian, since it is an approximation of the continuous Laplacian to the vertices of a graph and can be used to define and solve partial differential equations on a network. One such example is the heat equation on a network that has been extensively studied in the literature and has led to a number of important information processing algorithms. For example Zheng et al.^25^ have used the solution of a heat equation for anisotropic image smoothing. Sun et al.^26^ have demonstrated its applications for matching and retrieving three-dimensional shapes. Another example is the wave equation that has been proved useful in analyzing three-dimensional shapes^27^.

### Heat equation

The heat equation is a second order partial differential equation that describes the transfer of heat via conduction. It describes how the heat diffuses over time *t*, as it automatically flows from places where it is higher towards the places where it is lower. For a network, the heat equation is defined in terms of its Laplacian matrix as follows:

$$\begin{aligned} \frac{\partial H_t}{\partial t} = -L H_t, \end{aligned}$$

where *L* is the network Laplacian and $H_t$ is the fundamental solution of the heat equation called the Heat Kernel. The heat kernel can be thought of as the amount of heat that is transferred from one node *i* of the network to another node *j* in time *t* given a unit heat source at *i*. The heat kernel can be computed from the Laplacian matrix as follows:

3 $$\begin{aligned} H_t = \exp (-tL) = I-tL+\frac{t^2}{2!}L^2-\frac{t^3}{3!}L^3+\frac{t^4}{4!}L^4-... \end{aligned}$$

The heat kernel is a symmetric matrix of size equal to $n \times n$, where *n* is the number of nodes in the network. The (*i*, *j*)-th entry of the heat kernel can be computed from the eigenvalues and eigenvectors of the Laplacian matrix as follows:

$$\begin{aligned} H_t(i,j) = \sum _{k=1}^{n}\exp (-\lambda _k t)\phi _k(i)\phi _k(j). \end{aligned}$$

Suppose we inject a unit amount of heat at a node *i* of the network and allow it to diffuse through the edges connected to *i*. For an unweighted network, the heat will diffuse equally to all the neighbours of *i*. For a weighted network the diffusion rate is proportional to the weight of the edge. One of the useful properties of the heat kernel is that it is stable under small changes in the underlying network. Heat kernel can be interpreted as probability of Brownian motion to start at some node *i* and end at node *j* after time *t*. This means that $H_t(i,j)$ is a weighted average over all paths between the node *i* and the node *j* in time *t*.

It can be shown that the heat kernel is linked to different topological properties of the network for the different values of the parameter *t*. When $t \rightarrow 0$, then $H_t \simeq I-tL$, and therefore the heat kernel depends upon the local structure of the graph. In other words, for very small values of *t* (close to 0), the function $H_t(i,j)$ is mainly determined by the set of nodes that are directly connected to nodes *i* and *j*. As the value of *t* increases, this set grows and includes nodes that lie on the paths of length greater than two between nodes *i* and *j*. It is important to note that the rate of flow from node *i* to node *j* will depend upon the number of shortest paths between the two nodes. If the two nodes share many short paths of smaller length, diffusion between the two nodes will be fast. Therefore the heat will flow more rapidly among nodes in a more densely connected part of the network.

### Parametrised matrix forest index

In this paper we develop a framework for predicting missing links in a network that is based on the parameterised matrix forest index (PMFI) of the network. For this purpose, we first study the behaviour of PMFI for different values of its parameter $\beta$. The parametrised matrix forest index for parameter $\beta$, ($PMFI_{\beta }$) is defined as follows:

$$\begin{aligned} PMFI_{\beta } = \left( I+\beta L\right) ^{-1}, \end{aligned}$$

We first look at the case when the value of $\beta$ is small, in which case PMFI can be expanded as:

$$\begin{aligned} PMFI_{\beta } = I - \beta L + \beta ^2 L^2 - \beta ^3 L^3+... = \sum _{i=0}^{\infty }(-1)^i\beta ^iL^i. \end{aligned}$$

**Figure 1:**
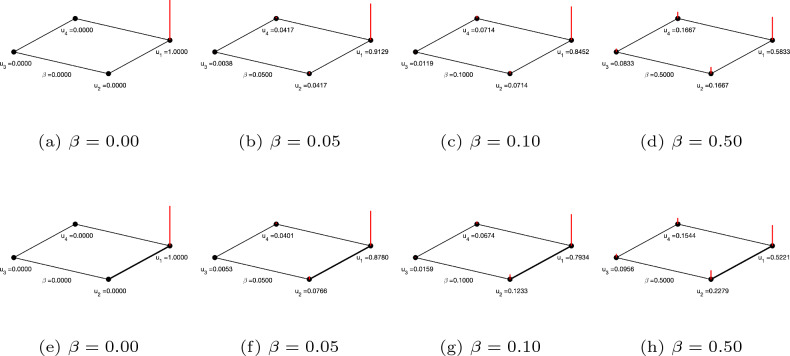
Approximation of heat diffusion process for small values of $\beta$.

Note that similarly to the heat kernel, when $\beta \rightarrow 0$, $PMFI_{\beta } \approx I-\beta L$. In literature, the PMFI is also referred to as regularised Laplacian kernel^28^ and can be used to simulate a heat diffusion process on a network. To demonstrate this, we take the examples of weighted and unweighted networks with four nodes and four links each. We set $H_t(i,j) = PMFI_{\beta }(i,j)$ and assume that the node $u_1$ has a unit heat. By increasing the value of the parameter $\beta$, we allow the heat to diffuse across the network. Figure 1 shows the simulation results for small values of $\beta$.

The top row of Fig. 1 presents the simulation results of an unweighted network with four nodes. Figure 1a depicts the initial configuration, where a unit heat is injected to node $u_1$. The amount of heat diffused to the remaining nodes of the network at time 0.05, 0.10, and 0.5 are presented in Fig. 1b,c,d respectively. Note that the node $u_1$ transfers equal amounts of heat to its immediate neighbours $u_2$ and $u_4$. However the amount of heat transferred to node $u_3$ is lower as it has to go through nodes $u_2$ and $u_4$. The bottom row of Fig. 1 shows the simulation results for a weighted graph. Here the weight of the edge $(u_1,u_2)$ is 2 while the remaining edge has a unit weight. As with the unweighted graph, we assume a unit heat at node $u_1$ (See Fig. 1e). It can be seen from Fig. 1f,g,h that the amount of heat transferred to the neighbour node $u_2$ is higher than the amount of heat transferred to the other neighbour node $u_4$. This is due to the fact that the diffusion rate is proportional to the weight of the links between the two nodes. The above discussion suggests that the value of $PMFI_{\beta }(i,j)$ can be thought of as the amount of heat transferred from node *i* to node *j* over time $t=\beta$, when $\beta$ is small. This amount will depend upon the number of paths of different length between node *i* and node *j*. Intuitively, this means, this value can be considered as a weighted average over all paths between the node *i* and the node *j*^26^, where the weights are determined by the length of each path. This motivates us to use $PMFI_{\beta }(i,j)$ with small values of parameter $\beta$ for predicting missing links in a network. By transforming the network into a weighted network using RA index, we show that the PMFI can predict links with a higher accuracy when compared to alternate methods.

Note that a special case of the PMFI is obtained when $\beta$ is set equal to 1. In this case the PMFI reduces to $\left( I+L\right) ^{-1}$ which is commonly known as Matrix Forest Index (MFI) in the literature. The value of MFI for the nodes *i* and *j* can be understood as the ratio of the number of spanning forests such that nodes *i* and *j* belong to the same tree to all spanning forests of the network. Due to its connections with global properties of the network, the MFI has been explored in the literature for predicting missing links in a network. For example in^29^ the authors have shown that, out of 20 local and global link prediction methods, the MFI gives second best performance when predicting missing links in a co-authorship network. The PMFI for other values of $\beta$ has also been used in literature to perform different tasks including predicting missing links in the network. For example, in^30^ the authors have tested PMFI with different values of $\beta$ to quantify the similarity between nodes in a bipartite network for collaborative recommendation tasks. However, the work reported here is different in two respects. Firstly, before applying the PMFI, we have transformed and pre-weighted the original adjacency matrix according to the Resource allocation index. This assigns a higher score to the pair of disconnected nodes that are exactly one hop away from each other. In this way, the underlying weighted matrix encodes information about the common neighbours of two disconnected nodes. Secondly, we have explored the PMFI both theoretically and empirically and, using synthetic and real-world examples, have shown why the proposed similarity index is different from other similarity indices such as resource allocation and matrix forest index when predicting missing links in a network.

### Proposed method

As discussed in the last subsection, for small values of $\beta$, $PMFI_{\beta }(i,j)$ is approximately equal to the amount of heat transferred from node *i* to node *j*, and therefore has the potential to be used as a measure for estimating the probability of existence of a link between a pair of disconnected nodes in a network. Following are some of the advantages of PMFI when used for predicting missing links in a network:

- When $\beta$ is small, the value of $PMFI_{\beta }(i,j)$ is determined by the set of shortest paths of smaller lengths between the nodes *i* and *j*. Therefore this index can capture the local structure of the graph around the nodes *i* and *j*. Note that, unlike the local similarity indices, such as CN, RA, and AA, PMFI, also consider paths of lengths greater than 2. However, since the heat diffuses at a slow rate through these paths, such paths are assigned less weight when predicting a link between two nodes.
- Since heat diffuses at a faster rate in densely connected areas of the network, PMFI index tends to predict the missing links within a community before predicting links between two different communities. A community is a subset of the network nodes that are more densely connected among themselves than the rest of the network. Therefore PMFI index can preserve the community structure of a network while predicting new links in the network.
- Since PMFI can potentially predict links between nodes that are more than two hops away, it can outperform local similarity indices when applied to the networks that do not follow the triadic closure principle and tend to form cycles of length greater than three, such as protein-protein interaction networks^22^.
- Finally, PMFI is robust to edge failure, when the two nodes are reachable through more than one path.

These characteristics of PMFI are further demonstrated through examples later in this section. Note that since the information diffuses more quickly in a densely connected area, one of the limitations of $PMFI_{\beta }(i,j)$ is that it tends to predict all the missing links in a densely connected community before predicting links in more sparse parts of the network. In order to increase the ability of PMFI to predict missing links in sparse communities, we convert the original network into a transformed weighted network whose link weight is determined by the adjacency matrix of the network and the information about common neighbours of the two nodes in the network. This is a two-step process. In the first step the network is transformed into a network where two disconnected nodes are connected by a link if they share at least one common neighbour. In the second step, the network is pre-weighted, where the weights are assigned by applying one of the local link prediction algorithms, such as CN, AA, and RA, to the original network (before the transformation). In this paper, we propose to use the RA index to weight the edges of the network as it outperforms other local similarity indices on many real-world networks^9^. We demonstrate in the experiment section that pre-weighting the adjacency matrix according to the RA index can result in improved performance. We refer to the resulting index as weighted parametrised matrix forest index (WPMFI).

**Figure 2:**
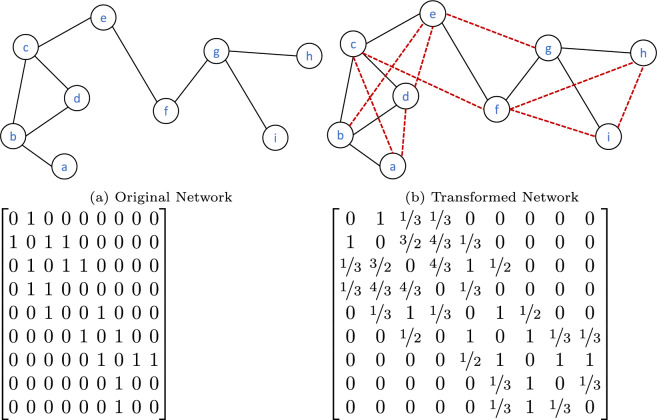
Original network and the transformed version of the network and their adjacency matrices.

Figure 2 demonstrates the process transforming and pre-weighting a network with 9 nodes and 9 links into a network with the same number of nodes and 18 links. The transformed network contains all the links that are present in the original network. In addition to this, two disconnected nodes in the transformed network are also connected by a link if they share at least one common neighbour. The link (*i*, *j*) is assigned the weight $w(i,j) = A(i,j)+RA(i,j)$, where *A* represents the adjacency matrix of the original network and *RA*(*i*, *j*) is computed from the degrees of the common neighbours of nodes *i* and *j*. More specifically, the value of *RA*(*i*, *j*) equals the sum of the reciprocal degrees of all the common neighbours of nodes *i* and *j*. Figure 2 shows the adjacency matrices of the original and the transformed networks. Consider the node *c* which is connected to node *b*, *d*, and *e* in the original network. Since *c* does not share any common neighbour with *e*, the weight of the link (*c*, *e*) equals 1. The nodes *c* and *b* share the common neighbour *d* with degree 2 and therefore the weight of the link (*c*, *b*) is $1+\frac{1}{2}=\frac{3}{2}$. Finally the weight of the link (*c*, *d*) is $\frac{4}{3}$ since *c* and *d* share a common neighbour *b* whose degree is 3.

**Figure 3:**
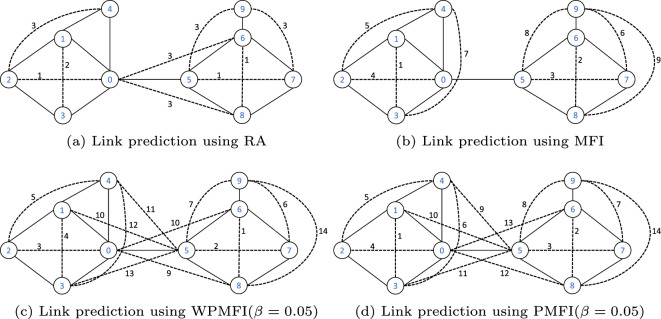
The order in which links are added by different indices in a small synthetic network. The edge label represents the rank of the added link.

In order to empirically highlight the characteristics and advantages of the WPMFI and compare it with alternative indices, we apply it, along the three other similarity indices (RA, MFI, and PMFI), to both a small synthetic network as well as a real world-network. The synthetic network consists of a network with 10 vertices and 12 edges and is shown in Fig. 3. The solid black lines represent the links of the original network, while the dashed lines show the links predicted by different indices. This network consists of two different communities, i.e., $\{0,1,2,3,4\}$ and $\{5,6,7,8,9\}$. Figure 3a shows the sequence (represented by the label of each predicted link) in which the links are added to the network according to the RA index. The RA index assigns the highest score to the pair of disconnected nodes (0, 2), (5, 7) and (6, 8). All three pairs of nodes have two common neighbours with degrees 2 and 3 respectively. Next the link (1, 3) is added which is followed by links (2, 4), (0, 6), (0, 8), (5, 9) and (7, 9) all having the same RA score. Finally, all the remaining pairs of disconnected nodes that are exactly one hop away from each other get connected.

In contrast to the local link prediction indices, such as RA index, the link prediction methods, based on the parametrised matrix forest index, predict links in order that respects the community structure of the network. In this example the first eight links predicted by all the three methods (MFI, PMFI, WPMFI) are the links that connect the nodes belonging to the same community. However, the order in which the links are predicted is different for all the three methods. The MFI may assign a higher score to a link between two nodes that are more than one hop away from each other. For example, before connecting the node pairs (0, 6) (and (0, 8)) that are one hop away from each other, the MFI index connects the nodes 8 and 9. The PMFI and the WPMFI, on the other hand, assign a lower score to the link (8,9). Both these methods connect the nodes that are exactly one hop away before connecting the nodes 8 and 9 that are two hop away. Note that, the PMFI and WPMFI differ by the order in which the links are added to the network. For the WPMFI, the order is more consistent with the RA index.

**Figure 4:**
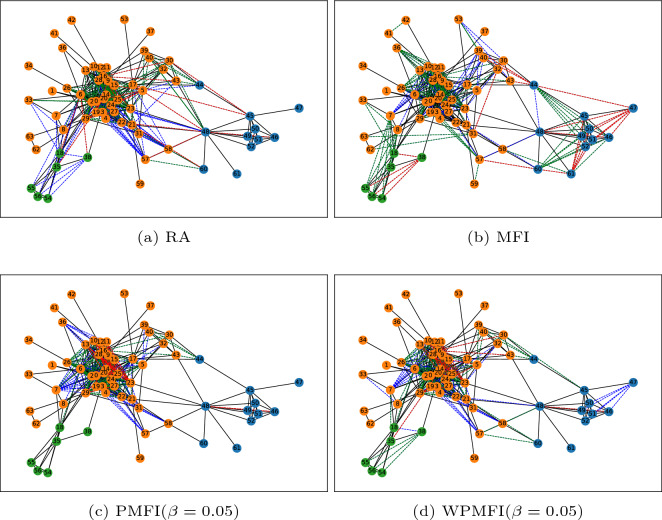
The order in which the new links are added to the network by different similarity indices.

We next consider a real-world network termed Bombing^31^. This network consists of 64 nodes and we have identified three different communities in this network using Girvan–Newman algorithm^32^. The nodes belonging to the same community are coloured identically to indicate community membership. As with the synthetic network example, we have applied all the four indices to predict new links in this network. For the RA index, we have used three different threshold values to predict a total of 149 new links in the network. These values are 0.25, 0.2 and 0.15 and they have resulted in 23, 84 and 42 new links in the network. Figure 4a shows the order in which the set of links are added by the RA index (dashed lines). The red, green and blue colours represent the first, second and the third set of links respectively. Figure shows that the RA index does not respect the community structure of the network. We note that all the three sets of links predicted by the RA index contain many links that are formed between nodes from two different communities. On the other hand, the order in which the links are predicted by the MFI index is different as shown in Fig. 4b. In this case the majority of the links in the first set are formed between the nodes belonging to the same community. For comparison purposes, we have shown the top 149 links in Fig. 4b (and also in Fig. 4c,d), where the first 23 predicted links are coloured as red, the next 84 as blue and the final 42 as green. In contrast to these two methods, the PMFI (for $\beta =0.05$) assigns higher scores to the links that could be formed between nodes belonging to the same community. However all the links, apart from two, across the three sets are formed between nodes that are members of the blue community (See Fig. 4c). Finally, as shown in Fig. 4d by pre-weighting the network using RA index, WPMFI is capable of predicting missing links in sparse communities and also respects the community structure of the network with only a small fractions of links predicted between nodes belonging to different communities.

In the experiment section, we have set $\beta =0.05$, when the proposed index is applied to real-world networks. We have demonstrated that PMFI achieves the best performance when $\beta$ is set to a value less than or equal to 0.05. As the value of $\beta$ increases, the performance of WPMFI decreases. It is worth mentioning that, when $\beta$ is very small, the order in which the first few links are predicted, by the WPMFI index, becomes similar to the order in which the links are predicted by the RA index. This is because when $\beta \rightarrow 0$, $WPMFI_{\beta } = I-\beta L = (I-D)+\mathcal{W}$, where $\mathcal{W}$ is the weighted adjacency matrix of the graph whose (*i*, *j*)-th weight is determined by RA index, if $(i,j)\notin E$ and $i\ne j$. For the Bombing example discussed above, if we set $\beta =10^{-4}$, the order in which the links are added by the WPMFI and the RA index is similar. But since WPMFI can potentially predict links between nodes that are more than one hop away, it can outperform RA index even when $\beta$ is small. However, the connection probability for nodes that are more than one hop away is very low in this case. Therefore, if links with very low connection probability are ignored, the performance of the PMFI for a very small value of $\beta$ will be the same as that of the RA index.

### Algorithm

The proposed algorithm for implementing WPMFI is given in Algorithm 1. This algorithm accepts the adjacency matrix $\mathcal{A}$ of the network and the value of the parameter $\beta$. Since we are investigating the behaviour for small values of $\beta$, we set its default value to 0.05. The output of the algorithm is the score matrix WPMFI, whose (*i*, *j*)-th entry is the likelihood score of occurrence of a connection between the nodes *i* and *j*. From Line-4 to Line-9, the algorithm first computes a matrix of scores computed according to the RA index. The diagonal values of the matrix are set to 0 in the loop from Line-10 to Line-12. Next, the weighted matrix of the original network is added to the score matrix which gives us the final weighted adjacency matrix $\mathcal{W}$ of the network. In Line-15, the Laplacian matrix $\mathcal{L}$ is computed which, along with the value of the parameter $\beta$, is used to obtain the final score matrix WPMFI.
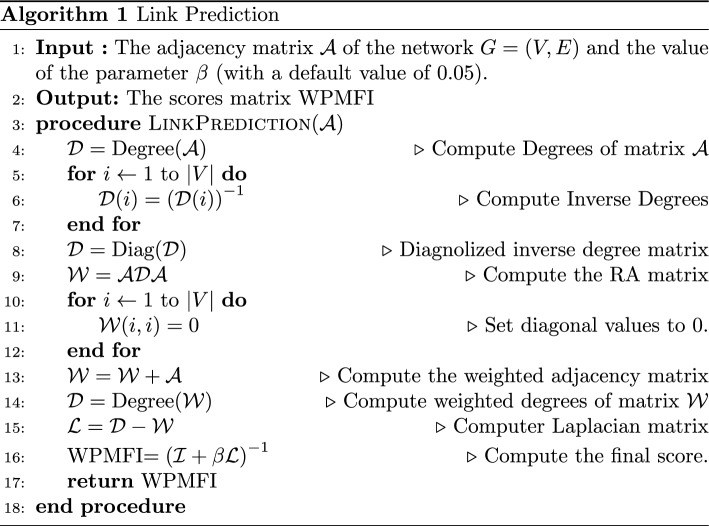


The running time of the Algorithm 1 is dominated by Lines 9 and 16. Line 9 requires two matrix multiplication operations and takes $O\left( |V |^3 \right)$ time in the worst case. Line 16 requires one matrix inversion operation which also takes $O\left( |V |^3 \right)$ time in the worst case. All the remaining operations either take $O\left( |V |^2 \right)$ time in the worst case or have linear worst-case running time. Therefore the worst case running time of Algorithm 1 is $O\left( |V |^3 \right)$. Note that in practice, the algorithm runs very fast. This is because of the fact that all the operations in the algorithm, including the two for loops, can be implemented using vectorised versions. In addition, since the matrix $\mathcal{A}$ is a sparse matrix and $\mathcal{D}$ is a diagonal matrix, the multiplications could be efficiently implemented using sparse matrix operations. Therefore, for large matrices, the running time of WPMFI is close to the running times of the alternative global link prediction algorithm such as Katz index and MFI.

## Experimental settings

### Evaluation metric

To estimate the performance of the proposed and alternative methods, we use the standard area under the receiver operating characteristic (*AUC*) metric^33^. To understand how this metric is computed, consider a simple undirected network $G = (V,E)$. We refer to the set of existing links *E* in the network as observed links while the remaining node pairs that are not connected are represented by the complement set $E'$. In other words, $E' = \{(u,v): u,v \in V, (u,v) \notin E\}$. Since the sets *E* and $E'$ are complement of each other, the two set are mutually exclusive and their union forms the universal set *U* that consists of all possible $\frac{n\left( n-1\right) }{2}$ links that the network *G* can have. To estimate the performance of the prediction algorithm, the set of observed links *E* is divided into two disjoint sets, namely, a training set $E^T$ and a probe set $E^P$. The metric AUC can be interpreted as the probability that a randomly chosen link in $E^P$ gets a higher score than a randomly chosen link in $E'$. The *AUC* is defined as follows:

$$\begin{aligned} AUC = \frac{\sigma _1+0.5\sigma _2}{\sigma }, \end{aligned}$$

where $\sigma$ is the number of independent comparisons, $\sigma _1$ is the number of times a missing link has a higher score than a non-existent link, and $\sigma _2$ is the number of times a missing link and a nonexistent link have the same score. If all the link scores are randomly generated according to an independent identical distribution then the value of AUC should be about 0.5. Therefore, a value greater than 0.5 indicates how well the prediction algorithm performs when compared to pure chance.

### Algorithms

To assess the performance of the proposed method, we compare it with a number of alternative methods. These include Common Neighbour (CN)^7^, Adamic Adar (AA)^8^, Resource Allocation (RA)^6^, Preferential Attachment (PA)^11^, Matrix Forest Index (MFI)^5^, Linear Optimisation (LO)^19^, Common Neighbour and Centrality (CNC)^12^, Common Neighbour and Distance (CND)^13^, Superposed Random Walk (SRW)^18^, Random Walk with Restart (RWR)^18^, Katz index^16^, and Local Path (LP)^20^. Some of these methods require additional parameters to be adjusted before we can apply them. The values of these parameters are set to the same values that are used in the original paper. Table 1 lists the mathematical formula for computing each of these indices.

**Table 1:** Formula for link prediction methods used in this paper. Here $\alpha$, $\beta$, *c* and *t* are the tunable parameters. $\Gamma _i$ represents the set of neighbours of node *i*, while $|\Gamma _i|$ represents the degree of the node *i*. $[A]_{ij}$ represents the (*i*, *j*)-th entry of the matrix *A*, $A^T$ represents the transpose of the matrix *A*, and $d_{ij}$ is the length of the shortest distance between node *i* and node *j*. *P* is the transition matrix whose (*i*, *j*)-th entry represents the probability that a random walker staying at node *i* will walk to *j* in the next step. It is computed as $P=D^{-1}A$. $\mathbf {e_i}$ is an $n\times 1$ vector with the *i*-th element equal to 1 and remaining are 0.

Method	Formula
Common neighbour	$CN(i,j) = |\Gamma _i \cap \Gamma _j |= [A^2]_{ij}$
Adamic adar	$AA(i,j) = \sum _{k \in \{ \Gamma _i\cap \Gamma _j \}}\frac{1}{\log |\Gamma _k|}$
Resource allocation	$RA(i,j) = \sum _{k \in \{ \Gamma _i \cap \Gamma _j \}}\frac{1}{|\Gamma _k|}$
Preferential attachement	$PA(i,j) = |\Gamma _i|\times |\Gamma _j|$
Matrix forest index	$MFI(i,j) = \left( I+L\right) ^{-1}$
Linear optimisation	$LO(i,j) = A\left( \alpha \left( \alpha A^TA+I\right) ^{-1}A^TA\right)$
CN and distance	$CND(i,j) = \frac{\left|\Gamma _i\cap \Gamma _j\right|}{2}+\frac{1-A(i,j)}{d_{i,j}}$
Superposed random walk	$SRW(i,j) = \sum _{l=1}^{t} \left( \frac{|\Gamma _i|\pi _{ij}(t)}{2|E |} +\frac{|\Gamma _j|\pi _{ji}(t)}{2|E|} \right)$ where $\mathbf {\pi _i}(t)=P^T\mathbf {\pi _i}(t-1)$
Random walk with restart	$RWR = q_{xy}+q_{yx}$ where ${q_x}=(1-c)\left( I-cP^T\right) ^{-1}{e_x}$
Katz index	$Katz(i,j) = \left( I-\beta A\right) ^{-1}-I$
Local path	$LP(i,j)=[A^2+\beta A^3]_{ij}$

### Datasets

The proposed method was tested on real-world networks that are obtained from diverse domains ranging from social networks to biological networks. These networks are publicly available and could be downloaded from KONECT^34^. Here we give a brief introduction of each of these datasets. Table 2 lists some of the topological properties of each of these networks.

*Karate*^35^ This dataset was collected from the members of a university karate club in 1977. This network is commonly known as the Zachary karate club and has been very popular for evaluating community detection and link prediction algorithms. The nodes of this network represent club members while the links represent ties between two members.

*Train bombing (Bombing)*^31^ This dataset is obtained from a list of 64 suspected terrorists who were believed to be involved in the Madrid train bombing on March 11, 2004. The nodes of the network are the suspected terrorists and a connection is established between the two nodes if the corresponding terrorists are friends or have co-participated in training camps.

*Iceland*^36^ This is a network of sexual contacts of male homosexuals in Iceland, collected in 1992.

*Les Misérables (LesMis)*^37^ This is an undirected network that represents the co-occurrences of characters in the famous ‘Les Misérables’ novel written by Victor Hugo. A node of the network represents a character of the novel and two nodes are connected if the two characters appeared in the same chapter of the book.

*Caenorhabditis elegans (Neurons)*^38^ This is a network of 279 neurons that represent the nodes of the network. Here the links represent the synaptic connections between neurons.

*E.Coli*^39^ This is a protein-protein interaction network of Escherichia coli that consists of 329 nodes and 456 links.

*Network Science*^40^ The nodes of this network represent scientists working on network theory and a link between two nodes indicates that the two corresponding authors are co-authors on the same paper.

*Infectious*^41^ This is a contact network of individuals who have attended the exhibition, “infectious: stay away” in 2009 in Dublin. The nodes of the network are individuals and a connection is established between the two nodes if there is a face-to-face contact between the corresponding individuals that was active for at least 20 seconds.

*Caenorhabditis elegans (Metabolic)*^42^ This is the undirected metabolic network of the roundworm Caenorhabditis elegans, where nodes represent metabolites (e.g., proteins), and links represent the physical interactions between them.

*US Air*^43^ This network encodes information about direct flights among 500 US airports. The nodes represent airports and two nodes are linked together if there is a direct flight between the corresponding airports.

*A Song of Ice and Fire (ASoIaF)*^34^ This is the fictional social network constructed from a series of fantasy novels ‘A Song of Ice and Fire’ by George R. R. Martin. The first volume of the series, A Game of Thrones, was published in 1996. The nodes of this network represent characters and a link between two nodes denotes that the two characters are mentioned within fifteen words of each other. This version of the dataset covers the first five books of the series.

*Email*^44^ This is a network of email communication between individuals at the University Rovira i Virgili in Tarragona in the south of Catalonia in Spain. Here the nodes represent individuals and a link is established between two individuals, if one of the two users has sent at least one email to the other user.

*Yeast*^45^ A protein-protein interaction network of yeast, where a node represents a protein and a link represents an interaction between the two proteins.

*Human Proteins (Vidal)*^46^ This network represents an initial version of a proteome-scale map of Human binary protein-protein interactions.

*Hamster*^34^ There are three different networks (Household, Friendship, Hamster) that have been obtained from an online social networking website, hamsterster.com. These Networks contain friendships and family-links between users of the website.

Note that some of these networks have been preprocessed for our purpose. For disconnected networks (networks having more than one connected component) only the largest connected component is considered and the statistics given in Table 2 are for the largest connected component. Some networks contain multiple links between the same pair of nodes and/or loops (links having the same source and destination) while in some cases edges are weighted. All the multiple edges, loops and weights were ignored in our case.

**Table 2:** Topological properties of the networks used in experiments. $|V |$ and $|E|$ are the number of nodes and links respectively. *C* is the clustering coefficient. $\langle k \rangle$ and $\langle d \rangle$ are average degree and average path length. Finally $\rho$ denotes the density of the network while *H* is the heterogeneity defined as $H = \frac{\langle k^2 \rangle }{\langle k \rangle ^2}$.

Datasets	$|V |$	$|E|$	*C*	$\langle k\rangle$	$\langle d\rangle$	$\rho$	*H*
Karate	34	78	0.588	4.588	1.204	0.139	7.769
Bombing	64	243	0.711	7.594	1.345	0.121	12.597
Iceland	75	114	0.614	3.04	1.6	0.041	8.36
LesMis	77	254	0.736	6.597	1.321	0.087	12.055
Neurons	279	2287	0.337	16.394	1.218	0.059	25.916
E.coli	329	456	0.222	2.772	2.421	0.008	12.314
Netscience	379	914	0.798	4.823	3.021	0.013	8.021
Infectious	410	2765	0.467	13.488	1.815	0.033	18.716
Metabolic	453	2025	0.655	8.94	1.332	0.02	40.098
US Air	500	2980	0.726	11.92	1.496	0.024	53.785
ASoIaF	796	2823	0.635	7.093	1.708	0.009	29.749
Email	1133	5451	0.254	9.622	1.803	0.009	18.688
Bible	1707	9059	0.71	10.614	1.688	0.006	41.629
Yeast	2375	11693	0.388	9.847	2.548	0.004	34.223
Vidal	2783	6007	0.109	4.317	2.42	0.002	15.776
Household	874	4003	0.317	9.16	1.608	0.01	34.176
Friendships	1788	12476	0.166	13.955	1.726	0.008	45.546
Hamster	2000	16098	0.573	16.098	1.794	0.008	43.776

The properties of the networks listed in Table 2 suggest that the networks used for the experiments have different topological structures. Some networks share similar properties. For example both the E.Coli and the Email networks share low clustering coefficient and low density. However the density of the Email is significantly higher than that of the E.Coli. This allows us to estimate the performance of the proposed method and compare it with alternative methods on structurally different networks.

## Results and discussion

In order to assess the performance of the proposed method we apply it and the alternative methods to the datasets discussed above. The performances are estimated using AUC. As discussed above, given a network $G=(V,E)$, we first partition its links into a training set, $E^T$ and a probe set $E^P$. The set $E^P$ is selected in such a way that the subnetwork obtained after removing the links belonging to set $E^P$ remains connected. This is done by first computing a minimum spanning tree (MST) of the original network *G*. An MST is a node connected network that does not contain any cycle. The set $E^P$ is then randomly selected from the *E* that does not belong to the MST. All the remaining edges, including the edges that belong to the MST, are then added to the training set $E^T$. The same training and probe sets are used for both the proposed and the alternative methods. In our first experiment, we use 90% of the observed links as for training and the remaining 10% for the test set. To obtain an unbiased estimate of performances, all the experiments were repeated 100 times for smaller networks (Karate, Bombing, Iceland, LesMis, Neurons, Netscience, Infectious, Metabolic, US Air, ASoIaF) and 10 times for larger networks (Email, Bible, Yeast, Vidal, Household, Friendships, Hamster). In each run of the experiment, an independent random sampling of the observed links into test and probe sets was performed. The average accuracies along with standard deviation values of each method on all the 18 datasets are reported in Table 3.

**Table 3:** AUC values for different datasets. The cell Bold shows the best performance while the one Italic shows the second best performance for each dataset.

Method	Karate	Bombing	Iceland	LesMis	Neurons	E.Coli
CN	0.7327±0.0890	0.9493±0.0288	0.9252±0.0517	0.9678±0.0209	0.8648±0.0123	0.6286±0.0652
AA	0.7704±0.0977	0.9572±0.0288	*0.9456*±*0.0455*	*0.9704*±*0.0210*	0.8800±0.0121	0.6327±0.0674
RA	0.7829±0.0986	*0.9582*±*0.0285*	0.9443±0.0450	0.9698±0.0211	*0.8862*±*0.0120*	0.6329±0.0675
PA	0.6970±0.1186	0.8014±0.0509	0.8519±0.0919	0.8286±0.0414	0.7281±0.0161	0.8388±0.0485
MFI	*0.7837*±*0.0682*	0.9413±0.0253	0.8883±0.0387	0.9481±0.0214	0.8755±0.0097	0.8890±0.0374
LO	0.6900±0.1140	0.9430±0.0366	0.9046±0.0562	0.3833±0.0222	0.7135±0.0239	0.6579±0.1011
CND	0.7412±0.0799	0.9530±0.0252	0.9291±0.0416	0.9691±0.0188	0.8659±0.0120	0.8612±0.0340
Katz	0.7745±0.0861	0.9470±0.0267	0.9300±0.0467	0.9668±0.0216	0.8721±0.0108	*0.8897*±*0.0356*
SRW	0.7496±0.1213	0.9236±0.0333	0.8700±0.0957	0.9575±0.0263	0.8463±0.0123	0.6026±0.0654
RWR	0.7240±0.0989	0.9130±0.0352	0.8945±0.0865	0.9531±0.0265	0.8207±0.0126	0.6051±0.0617
LP	0.7799±0.0839	0.9467±0.0271	0.9314±0.0449	0.9672±0.0211	0.8732±0.0107	0.8739±0.0469
WPMFI	**0.8249**±**0.0619**	**0.9629**±**0.0331**	**0.9526**±**0.0291**	**0.9757**±**0.0166**	**0.9002**±**0.0097**	**0.9011**±**0.0301**

Method	Netscience	Infectious	Metabolic	US Air	ASoIaF	Email
CN	0.9904±0.0065	0.9554±0.0073	0.9283±0.0108	0.9664±0.0054	0.9715±0.0052	0.8763±0.0123
AA	0.9922±0.0065	0.9586±0.0073	0.9536±0.0095	0.9715±0.0050	0.9752±0.0052	0.8780±0.0122
RA	0.9922±0.0064	0.9593±0.0074	*0.9572*±*0.0092*	*0.9744*±*0.0048*	0.9754±0.0052	0.8775±0.0122
PA	0.6053±0.0422	0.7237±0.0149	0.7957±0.0230	0.9282±0.0121	0.8777±0.0127	0.8142±0.0099
MFI	0.9924±0.0027	0.9669±0.0031	0.9181±0.0093	0.9451±0.0047	0.9566±0.0041	0.9317±0.0065
LO	0.9838±0.0145	0.8352±0.0202	0.3866±0.0118	0.7448±0.0215	0.3835±0.0113	0.3205±0.0074
CND	*0.9934*±*0.0023*	0.9674±0.0042	0.9291±0.0105	0.9676±0.0048	0.9761±0.0037	0.9209±0.0080
Katz	0.9915±0.0026	*0.9703*±*0.0035*	0.9274±0.0108	0.9659±0.0056	0.9761±0.0037	*0.9364*±*0.0062*
SRW	0.9581±0.0163	0.9462±0.0073	0.8954±0.0135	0.9602±0.0066	0.9585±0.0066	0.8661±0.0142
RWR	0.9586±0.0154	0.9437±0.0072	0.8759±0.0132	0.9569±0.0066	0.9538±0.0063	0.8712±0.0127
LP	0.9916±0.0028	0.9700±0.0038	0.9281±0.0107	0.9664±0.0054	*0.9764*±*0.0037*	0.9325±0.0070
WPMFI	**0.9961**±**0.0022**	**0.9773**±**0.0032**	**0.9588**±**0.0092**	**0.9770**±**0.0039**	**0.9833**±**0.0033**	**0.9414**±**0.0062**

Method	Bible	Yeast	Vidal	Household	Friendships	Hamster
CN	0.9816±0.0024	0.9324±0.0039	0.6750±0.0125	0.9018±0.0098	0.8206±0.0030	0.9722±0.0020
AA	0.9879±0.0024	0.9330±0.0039	0.6758±0.0125	0.9068±0.0100	0.8230±0.0029	0.9754±0.0020
RA	*0.9887*±*0.0024*	0.9331±0.0039	0.6758±0.0125	0.9061±0.0098	0.8235±0.0029	0.9766±0.0019
PA	0.8270±0.0101	0.8867±0.0053	0.8590±0.0068	0.9179±0.0048	0.8899±0.0059	0.8474±0.0075
MFI	0.9728±0.0024	0.9811±0.0038	0.8967±0.0062	0.9027±0.0101	*0.9472*±*0.0020*	0.9708±0.0020
LO	0.4438±0.0035	0.4126±0.0063	0.2412±0.0096	0.2490±0.0099	0.2108±0.0044	0.4330±0.0043
CND	0.9832±0.0021	0.9807±0.0035	0.8849±0.0078	0.9185±0.0089	0.8819±0.0021	0.9786±0.0014
Katz	0.9803±0.0017	*0.9838*±*0.0036*	*0.9045*±*0.0079*	*0.9356*±*0.0092*	0.9370±0.0023	0.9808±0.0012
SRW	0.9533±0.0049	0.9276±0.0032	0.6384±0.0157	0.8964±0.0104	0.8242±0.0031	0.9679±0.0022
RWR	0.9471±0.0038	0.9283±0.0031	0.6637±0.0134	0.8957±0.0090	0.8184±0.0034	0.9592±0.0022
LP	0.9805±0.0017	0.9808±0.0035	0.8596±0.0124	0.9356±0.0093	0.9380±0.0021	*0.9809*±*0.0013*
WPMFI	**0.9916**±**0.0016**	**0.9877**±**0.0031**	**0.9153**±**0.0051**	**0.9458**±**0.0073**	**0.9613**±**0.0017**	**0.9855**±**0.0014**

It is important to note that the alternative methods were chosen in such a way that all the three categories of algorithms, namely the local, the global and the quasi-local link prediction algorithms, are included in the comparison. In Table 3, the first four methods CN, AA, RA and PA are local link prediction algorithms, the next four methods MFI, LO, CND, and Katz are global link prediction algorithms, and the next three methods SRW, RWR, and LP are the quasi-local link prediction algorithms. There are a number of important observations that can be made from the results shown in Table 3. Firstly, we note that the global link prediction does not always give best performance when compared to the local and the quasi-local methods. For example, for the Iceland and the Metabolic datasets the AA and the RA indices give significantly better performance when compared to the global methods such as Katz index. Secondly, since most of the local link prediction indices are based on the triadic closure principle (TCP), they fail to predict true links in networks with very low average clustering coefficients. For example, from Table 2, we can see that the three networks Vidal, E.Coli, and friendship have very low clustering coefficients and all the local TCP-based link prediction algorithms give poor performance on these datasets. On the other hand, these TCP-based methods perform very well and may achieve better performance when compared to global and quas-local methods when applied to datasets with very high clustering coefficients. This is evident when we compare the performances of all the methods on networks with high clustering coefficients such as Bombing, LesMis, Netscience, US Air and Bible. Finally, it is evident from the table that the proposed link prediction index, *WPMFI*, outperforms all the alternative methods on all the datasets. It does not only give better results for networks with low clustering coefficients but it also improves the performances of local link prediction algorithms on networks with very high clustering coefficients such as E.Coli, and Vidal. It also gives better performance when compared to the MFI index applied on the original network.

**Figure 5:**
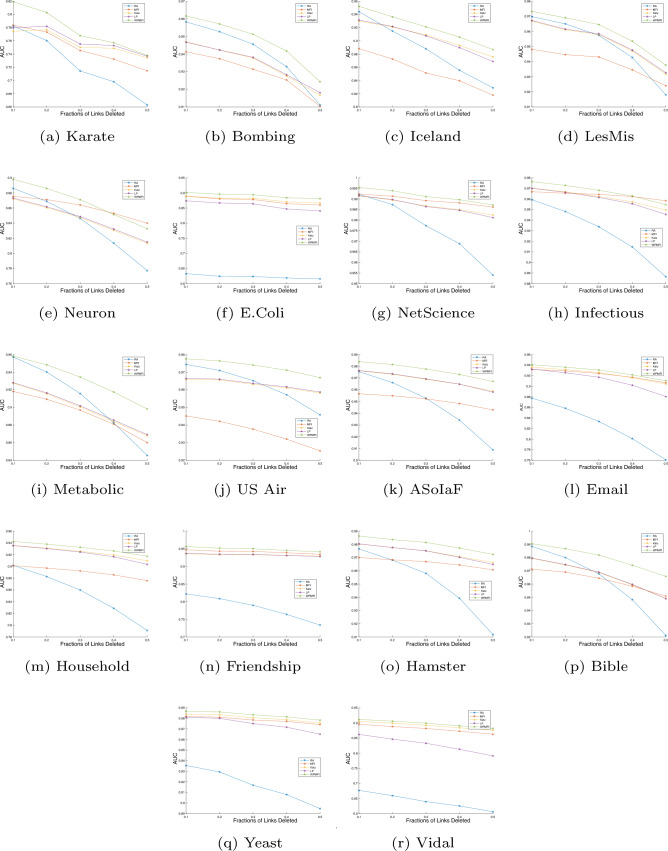
The accuracies estimated by AUC with different split of train/probe sets. All the experiments are executed multiple times and average values of AUC are executed.

We next investigate the performance of the proposed method and the other methods with varying sizes of the training set. For this purpose we randomly choose 10%, 20%, 30%, 40%, and 50% links for the probe set respectively, while the remaining links are used for the training set. As with the previous experiment, the probe set $E^P$ is chosen in such a way that the network obtained after removing links $E^P$ remains connected. For computational reasons, we have chosen only four alternative methods for comparison with at least one candidate method from each of the three categories of link prediction algorithms. These include RA (local), Katz (global), LP (quasi local), and the MFI. The experiments were repeated 100 times for smaller networks and 10 times for larger networks and in each run of the experiment, an independent random sampling of the observed links into test and probe sets was performed. The performances were estimated using AUC and the average values of AUC of the proposed and other state-of-the-art methods are plotted in Figure 5.

These results show that WPMFI outperforms other methods when we reduce the size of the training set $E^T$. We note that the performance of the RA index decreases significantly with decrease in training size. This is due to the fact that when more edges are removed from the network the local topology of the network around a node is likely to be changed. Therefore there is a sharp decline in the performance of the RA index when the number of training links are decreased. However, the WPMFI can still predict missing links with higher accuracy. For example for Metabolic, US Air, and Bible datasets the performance of RA is close to that of WPMFI when 90% of the links are used for the training set. However the performance of RA is significantly less than that of WPMFI when only 50% of the links are used for the training set. This suggests that WPMFI can still give reasonably good performance when very limited information is available about the structure of the network.

**Figure 6:**
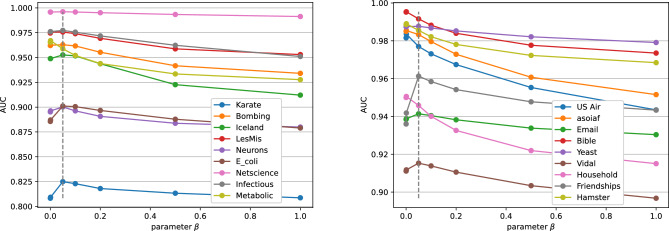
Effect of the parameter $\beta$ on AUC values. The values of $\beta$ are chosen as 0.0001, 0.001, 0.05, 0.1, 0.2, 0.5, and 1. The dashed gray vertical line indicates the value of 0.05 that was chosen in the experiments.

In order to analyse the importance of the parameter $\beta$, we apply WPMFI multiple times by varying the value of parameter $\beta$ to all the real-world networks. We have tested the performance of WPMFI by choosing seven different values of $\beta$ from the set $\{0.0001, 0.001, 0.05, 0.1, 0.2, 0.5, 1\}$. Figure 6 shows the AUC values for all the 18 datasets. For better visualisation, the results are presented in two separate figures. These results clearly demonstrate that we achieve better performance, when the value of the parameter $\beta$ is kept low. There are two important observations to note from Fig. 6. Firstly, in most cases, the performance tends to decrease monotonically as the value of $\beta$ increases from 0.05 to 1. This suggests that it is not useful to set the value of $\beta$ greater than 0.05. Secondly, the performance of WPMFI does not always improve when $\beta$ is further reduced and set to a smaller value less than 0.05. We note that in few cases the performance increases but in other cases it either reduces or remains roughly the same. We also note that the difference in the performance when $\beta =0.0001$ and when $\beta = 0.001$ is negligible in most cases. As discussed in Sect. 3.4, when $\beta$ is very small PMFI does not respect the community structure of the network as the link predicted by PMFI in this case will tend to follow the order in which the links are predicted by the RA index. We therefore set $\beta =0.05$ in all our experiments.

**Figure 7:**
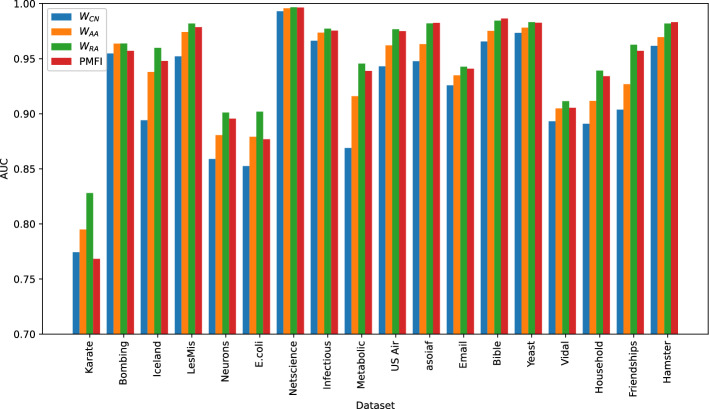
Comparing PMFI without and with pre-weighting. Here PMFI represents the parametrised matrix forest index applied to the original adjacency matrix without transformation and pre-weighting, while $W_{CN}$, $W_{AA}$, and $W_{RA}$ represent WPMFI when the original adjacency matrix is transformed and pre-weighted according to CN, AA, and RA indices respectively.

In our final experiment, we analyse the performance of WPMFI by using different methods for pre-weighing the adjacency matrix of the transformed network. For this purpose, we first transformed the network by connecting two disconnected nodes if they share at least one common neighbour. Next, we have pre-weighted the adjacency matrix of the transformed network according to CN, AA, and RA indices. We have applied the resulting indices to all the 18 datasets. In addition to this, we have also applied PMFI to the original adjacency matrix (without transforming and pre-weighting the original adjacency matrix). The value of the parameter $\beta$ was set to 0.05 in all cases. The AUC values for all the four methods are shown in Fig. 7. These results show that in most cases the parametrised matrix forest index pre-weighted with RA index gives superior performance when compared to all the other versions while in very few cases it gives comparable performance to PMFI. This demonstrates that the performance of PMFI can be improved by pre-weighting the matrix according to the RA index.

## Conclusion

In this paper we have investigated the applications of parametrised matrix forest index for predicting missing links in a network. We have demonstrated that the parametrised matrix forest index is related to a heat diffusion process on a network and therefore has the potential to be used for predicting missing links in a network. The edges of the networks are weighted in a manner such that the information flows more quickly between two nodes that are highly likely to be connected in near future than the two disconnected nodes that are less likely to be connected. This was achieved using the popular resource allocation index. The method was tested on 18 different real-world networks and was compared to other state-of-the-art methods under different settings. Although the proposed approach was tested only on unweighted networks, one of the advantages of the work presented here is that it can be easily extended to weighted networks. In terms of future work we plan to extend the work presented here to more complicated cases such as bipartite networks and multiplex networks and explore its applications in real-world networks such as drug-target interaction networks.

## Data Availability

The datasets analysed during the current study are publicly available in the Konect repository, and can be downloaded from http://konect.cc/networks/.
